# Interprofessional communication in the emergency department: residents’ perceptions and implications for medical education

**DOI:** 10.5116/ijme.5bb5.c111

**Published:** 2018-10-25

**Authors:** Marleen Olde Bekkink, Susan E. Farrell, James Kimo Takayesu

**Affiliations:** 1 Radboud University Medical Centre, Department of Internal Medicine, Nijmegen, Netherlands; 2 Massachusetts General Hospital, Institute of Health Professions, Center for Interprofessional Studies and Innovation, Boston, USA; 3 Massachusetts General Hospital, Department of Emergency Medicine, Boston, USA

**Keywords:** Interprofessional communication, interprofessional collaboration, resident training, patient safety

## Abstract

**Objectives:**

Objectives of the current study were to: i) assess residents’ perceptions of barriers and enablers of interprofessional (IP) communication based on experiences and observations in their clinical work environments, ii) investigate how residents were trained to work in IP collaborative practice, iii) collect residents’ recommendations for training in IP communication to address current needs.

**Methods:**

Focus group study including fourteen Emergency Medicine (EM) residents, who participated in four focus groups, facilitated by an independent moderator. Focus group interviews were audiotaped, transcribed verbatim, independently reviewed by the authors, and coded for emerging themes.

**Results:**

Four themes of barriers and enablers to IP communication were identified: i) the clinical environment (high acuity; rapidly changing health care teams, work overload, electronic communications), ii) interpersonal relationships (hierarchy, (un)familiarity, mutual respect, feeling part of the team), iii) personal factors (fear, self-confidence, uncontrolled personal emotions, conflict management skills), and iv) training (or lack thereof). Residents indicated that IP communication was learned primarily through trial and error and observing other professionals but expressed a preference for formal training in IP communication.

**Conclusions:**

Based on this pilot study, barriers to effective IP communication in the ED were inherent in the system and could be exacerbated by relational dynamics and a lack of formal training. Opportunities for both curricular interventions and systems changes were identified and are presented.

## Introduction

Interprofessional (IP) communication describes the sharing of information (by means of verbal, writing or other medium) among members of different health professionals to influence patient care positively. This includes communication that may either be intentional or unintentional. In a busy, high-acuity, academic emergency department (ED), successful IP communication between residents and non-physician staff is crucial for safe and effective collaborative patient care. Poor IP collaboration is associated with inefficient patient care,^1^ a higher prevalence of medical errors^2^^,^^3^ and lower job satisfaction.^4^ Successful IP collaborative practice is highly dependent on effective IP communication. IP communication is one of the core competency domains for successful IP collaborative practice.^5^ In a high-acuity, academic emergency department (ED), communication between residents and non-physician staff is crucial for safe and effective patient care. This high-interaction workplace creates large communication loads on clinicians.^6^ Poor training and a high level of interruptions may induce cognitive overload and result in impaired communication, clinical performance and patient safety.^7^^,^^8^

The World Health Organization (WHO) has called for the implementation of IP training in medical curricula,^9^ stating that students need to understand how to work in an IP collaborative practice before they are ready to enter the workplace as a member of a health care team.^9^ Residents are often expected to act as the leader of a team even though their experience may be far less than other team members. An additional barrier to IP communication is that teams in emergency settings are often ad hoc, with varying team members who may not have worked together before, nor are likely to work in the same team composition again.^10^ As these teams cannot rely on prior collaborative work experience, explicit communication is needed to achieve optimal group performance. Curricular interventions are needed to prepare residents to work in such highly demanding settings. The principle of the Crew Resource Management (CRM) concept used in aviation upholds that communication and coordination behaviors are identifiable and teachable.^11^^,^^12^ These lessons are applicable to other high-risk environments such as health care organisations.^13^

Residents compose a large part of the workforce in academic health care systems, delivering a large portion of direct patient care under the supervision of attending physicians, bridging the gap between patients, the attending physician, nursing and allied health professionals. Because of their central role in the health care team, residents are likely to influence team functioning and patient outcome. Therefore, capturing their perspectives and needs is a key step in improving health care team functioning. As residents are still in a position of training and developing their knowledge and skills, optimizing their training in IP communication is a high-value target to improve collaborative practice and quality of care.

This study aimed to explore EM residents’ perceptions and behaviors related to IP communication in order to formulate training methods to enhance these skills.  Specifically, the objectives were to i) assess residents’ beliefs and perceptions of barriers and enablers to IP communication based on experiences and observations in their clinical work environments, ii) investigate how residents were trained for IP collaborative practice, iii) to collect residents’ recommendations for training in IP communication.

## Methods

### Participants and setting

Participants were EM residents from an academic EM training program (Year 1– 4, Harvard-Affiliated Emergency Medicine Residency at Massachusetts General and Brigham & Women’s Hospitals). All EM residents in the training program (n=60) received an email invitation to participate in the focus groups. No incentives were offered. The focus groups were to be held at convenient times based on routinely scheduled teaching conferences in both teaching hospitals. The study purpose and consent procedure were included in the email invitation and again prior to the start of the focus group. The participants gave verbal consent for audio-recording prior to the focus group discussion. Fourteen residents (23.3%) agreed to participate in one of the four focus groups. The male to female ratio was 1.1. Residents from each year were represented in the focus groups. Demographics are presented in Table 1. Ethics approval was obtained from the Institutional Review Board of the Massachusetts General Hospital and the Brigham and Women’s Hospital. Participation was on a voluntary basis, and residents were assured they could stop participation at any time. Participation was voluntary and confidential.

**Table 1:** Characteristics of the focus groups and participants (N=16)

Focus group	1	2	3	4
Number of participants^*^	4	2	5	5
Average age	29	31	30	33
Male: female	3:1	0:2	3:2	2:3
Year of training				
PGY1	1	-	4	2
PGY2	-	1	-	2
PGY3	1	-	-	-
PGY4	2	1	1	1
Duration (min)	75	20	104	60

*The total number adds up to sixteen. Two residents participated in two focus groups. A total of fourteen residents participated in the study.

### Data collection

The focus group study included four focus groups that lasted 20-104 min (average: 65 min, total 259 minutes). Focus groups were guided by an independent moderator (MOB). Interview questions regarding IP communication facilitated the dialogue (see Appendix A). The interview questions were based on prior literature research. Independent review by EM educators validated the questions. All interviews were audiotaped and transcribed verbatim. Participants’ demographic data were collected after each focus group. Names of participants were replaced by codes to ensure privacy and confidentiality.

### Data analysis

The data analysis was initiated by using an open coding process. No a priori codes were used. An inductive content analysis approach was used for coding and development of themes.^14^ Open coding was performed independently by all study staff members (JKT, SEF, MOB). Subsequently codes were grouped in themes and subthemes. Themes were then iteratively revised using in-depth discussions to explore relationships among the themes.^15^ Thematic saturation was reached after four focus groups. Verification of thematic analysis with participating subjects was performed through the delivery of a compilation of themes. The participants approved the themes without modifications. There were no changes made after this step.

**Table 2:** Barriers to IP communication separated into four major themes

Themes	Subthemes	Quotes
I. Clinical environment (system)	Work overload	*“I think in intern years you are literally asked to do impossible things. It is not possible to meet everyone’s expectations, so you are always disappointing people. That is part of why it is so frustrating. Your workload is literally not possible. So you start choosing in which ways you take shortcuts.”*
	High acuity setting/ time pressure	*“Time is the biggest challenge. Taking the time to come up with cogent questions or dialogue and having the time to clear up any questions. Usually it is: I need this, I need this, I need this.”*
	Rapid changing healthcare teams	*“It so hard to attain familiarity with this enormous staff at every different location, on every different shift and then people are switching all the time. It makes it really disconnecting”*
	Electronic order entry	“*A lot of times it’s pretty easy, especially when it’s busy, to run out of the room an write your orders and never talk to the nurse.”* *“The only times that I got in trouble, was when I was just throwing in orders and they were not done because I never communicated them to anyone as to why we were doing them.”*
II. Interpersonal relationships	Mutual respect	*“A lot of the times where discussions have gone wrong, is where you didn’t care about the person’s name, you don’t care about how busy they are, or what they have to do, or you devalue or invalidate their opinion because you feel like you’re better trained just as a function of like ten year or the letters you have behind your name.”*
	Hierarchy	*“I do think especially as a med student, and as a new resident, there tends to be a negative hierarchy, where your opinions are not respected in an appropriate way.”* *“Something I was very aware of was how others really interacted with regards to their understanding of their position on the totem pole.” * *“There’s some built-in antagonism between nursing and physicians, which is based on their roles and I don’t think we’ll ever be able to dissipate.”*
	Unfamiliarity	*“We’re not familiar with any of the training that either our PCA’s or nurses, or respiratory therapists go through”* *“I still have problems where I don’t recognize what other peoples’ workflows are, and usually that’s when I still have conflicts when I am not appreciating what someone else’s priorities or attention is at that moment and I interject something sort of without realizing that I’m doing.”*
	Feeling part of team	*“Seeing the care team as a team, especially with the nurses, by going back and updating them on the plan so that they feel that they are updated and not just getting the computer orders.” *
III. Personal factors	Self confidence	*“I am not confident enough in myself yet that I feel comfortable a lot of times approaching anyone, but especially anyone more senior than I am.”*
	Fear	*“I think everyone has an intense fear of looking stupid or looking dumb. And that really prevents a lot of the communication”*
	(Uncontrolled) personal emotions	*“She was frustrated, and I was frustrated, and I was just like: “Aargh”, “What do you do?!” I snapped at her, and then I left, but then I thought: “That’s terrible.” * *“I think that the biggest place I saw yelling was in the operation room from the attending surgeon to the scrub nurses, I noticed that the nurses would actually hide things from that surgeon.”*
	Conflict management skills	*“Always connect the conversation that you have to the patient, so you combine interests.”* *“I think trying to find some sort of common point in discussion. I try to step back and try to realign the conversation to something everyone can agree upon doing.”*
IV. Training	Lack of formal training	*“We don’t have any obviously prior training in this area at all. I think that’s one of the reasons that we should. We are essentially trialed by fire since the day we start, especially as an intern.”*
	Lack of formal feedback/ debrief sessions	*“I think we tend to talk about our communication with nurses, without actually ever including them in the conversation.”* *“Right now, there isn’t much feedback. There is no formalized feedback.”*
	Lack of role models	*“The lack of training left you looking to role models, and unfortunately most of the role models that we have are usually people who are the loudest, most assertive and maybe more aggressive end up in getting their job done. And then, in the hospital that sort of disruptive behavior is acceptable as long as the task is accomplished.”*
	Learning by trial	*“Almost all of my communication skills that I learned in residency, I have learned from doing things wrong first, and then later I learned how to do it the right way”*

## Results

**Figure 1:**
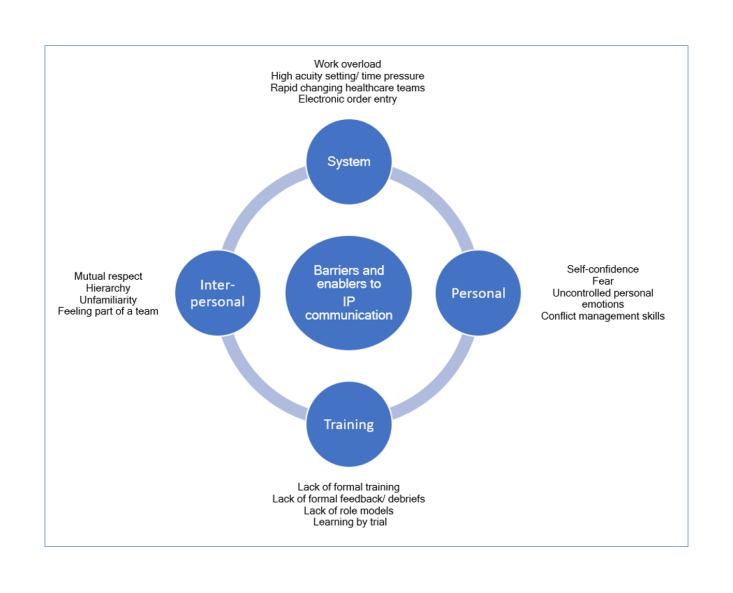
Four major themes relating to IP communication

Four major themes that impact IP communication were identified: i) the clinical environment, ii) interpersonal relationships iii) personal factors, and iv) training (or lack thereof) (Figure 1). Barriers and enabling factors in each of the four major themes are discussed. Table 2 lists a summary of major themes, including subthemes and representing quotes.

### The clinical environment

Time pressure was seen as a significant challenge to IP communication, inherent to the high acuity setting of the ED and the constant high volume of workflow as a result of the rapid patient turnover. Time pressure hampers residents’ abilities to communicate their thought processes behind requests and orders, although they acknowledged the importance of communicating their thought process in order to: i) increase other team members’ understanding of critical problems ii) acknowledge other professionals’ skills and expertise and iii) increase other professionals’ motivation as members of the care team, as illustrated by the following quote:

“One of the things which has made a big difference to my nursing communication is to ask nurses: “What do you think is going on and what do you think we should be doing?” Or when I do have some ambiguity to say: “I am sort of on the fence about this particular decision. What does your experience tell you about this instance, and how can we use your insight to help make a decision here?” And I found that, this has really dramatically improved my relationships with the nursing staff, to get them involved. And they also become stakeholders in the decision-making process and then, once that’s the case, then I think they’re more interested in seeing what the outcome is.” (Male resident, PGY2)

Another barrier to IP communication identified in the current clinical learning environment was the rapidly changing care teams that diminish relationship building. Residents frequently change hospitals and rotate through different clinical teams, hampering relationship building. Residents indicated that knowing other professionals’ names and expertise facilitates better patient care by facilitating closed-loop communication; however in practice, residents work for short periods of time with the same people. These changes in team composition also limit residents’ understanding of others work-related needs and how other professionals’ individual roles complement their own. Complicating this fact, residents commented on how electronic ordering systems have fostered rapid digital ordering, limiting the need for face-to-face interaction and discussion.

### Interpersonal relationships

Interpersonal relationships influence IP communication. Residents indicated that traditional hierarchical professional boundaries still exist. This included hierarchies at multiple relationship levels: the physician-nurse relationship; inexperienced intern - experienced nurse relationship; nurse - medical student relationship. As an example, one resident cited that working with an experienced nurse as an inexperienced intern poses challenges to effective IP communication, as illustrated by the following quote:

“Basically the nurse was telling: “I’ve been doing this for longer than you have been alive probably. What year were you born?” How am I supposed to- I mean, that is a very demeaning comment to say. How are you supposed to respond to that? It’s puts you into your place. You’re there; all of a sudden you’re isolated.” (Male resident, PGY 2).

The power differentials that exist among different professionals on the health care team in a training environment can be significant, hampering feelings of inclusivity and connection. Team building is further impeded by unfamiliarity both on a content level (unawareness of the other professionals’ skillset and expertise) and a relationship level. Residents indicated that having a healthy professional relationship positively influences collaboration. The building of such a relationship is fostered by expression of mutual respect, evidenced by listening to individual opinions and acknowledging their expertise and contribution to the team. It helps to include the other professionals in the decision making and updating them on the plan as illustrated by the following quote:

“Like trying to circle back and touch base at least a few times throughout a patient’s course. So that everybody knows what is going on. And I think that that has helped me to be more accountable for my patients and also to make sure that the team is all on the same page.” (Female resident, PGY2)

### Personal factors

Personal factors, such as one’s knowledge and experience increase confidence and self-esteem, subsequently impacting IP communication. Many residents perceived barriers to IP communication when explicitly expressing a lack of knowledge, while others indicated that being open about a knowledge gap enabled communication, rather than acting as a barrier.

“I think admitting when I don’t know things helps a lot, especially with nursing staff. Telling them, you know: this is why I haven’t put in orders yet, or this is why I haven’t decided yet. I don’t know. I need to figure it out.” (Female resident, PGY 1)

Other residents indicated they are sometimes reluctant to express knowledge or skill gaps for fear of looking incompetent in front of other professionals. Interns, in particular, experience difficulties in approaching other professionals and hesitate to speak up because they feel insecure about their own level of knowledge and expertise. While a nurse’s experience may far exceed their own, residents feel legally responsible for diagnostic and therapeutic decision-making, resulting in gaps in communication and mutual understanding.  Moreover, the fear of being misperceived is not limited to interns and junior residents. More senior residents indicated that fear never really goes away, as illustrated by the following quote:

“If I am in a situation where I see that somebody has done something that isn’t standard and maybe needs corrective action, there is always an intense fear that I feel: do I go over and correct them? For the sake of being too aggressive? And hurting their feelings? Or the fear of: oh, I didn’t say enough, so now I’ve missed a teaching opportunity for this person and now everybody else thinks I am a pushover.” (Male resident, PGY 4)

Additionally, resident workload pressures and responsibilities sometimes resulted in emotional exhaustion making residents more susceptible to expressing uncontrolled personal emotions towards other professionals, hampering effective IP communication.

Another relationship subtheme that emerged related to conflict management skills (exploration and de-escalation). When interpersonal conflicts arise, residents learned to manage it through individual experience rather than formal training.  Residents described de-escalation by focusing conflicted conversations on patient-centered, common goals. Exploring the situation through open questioning with an unprejudiced attitude helped to identify underlying communication problems and steer the process in a conflict-neutral direction. Expressing empathy and “stepping into the other person’s shoes” were expressed as mutually beneficial to reconnecting interpersonally and fostering IP communication.

### IP communication training in the formal curriculum

Residents indicated that IP communication skills were learned primarily through observation of others but expressed a preference for this topic as part of their formal curriculum. The most common source of learning was by trial and error. If residents did not achieve their desired goals, they would learn from the encounter and change their approach the next time they encountered a similar experience. Residents also indicated direct observation could be a major element of learning. However, they identified a lack of champions who value and model effective IP communication as a means to level power differentials to prevent conflict that detracts from high-quality patient care.

### Recommendations for future training

Residents proposed different learning formats that they thought might be beneficial for training in IP communication. These formats include i) IP simulations with debrief, ii) case discussions and iii) literature on basic communication and leadership skills. Interdisciplinary simulation training was preferred because it is a safe environment in which residents can practice together and debrief with immediate feedback on collaborative behaviors and attitudes. Additionally, residents suggested discussing paper cases and complications from IP communication failures (as in Morbidity & Mortality conferences) in IP groups with a particular focus on how poor communication has affected a patient outcome with suggestions for future improvements. Different IP perspectives could provide an opportunity to learn about other professionals’ informational and communication needs. Finally, residents suggested including literature reviews on communication and leadership, supplemented by IP small group discussions on the application of theory to daily practice.

## Discussion

Our results indicate that barriers to effective IP communication persist in daily practice in an academic ED. Barriers were identified at multiple levels, including the clinical environment (system level), interpersonal relationship level, and individual level. These findings are consistent with a recent review of the current literature.^16^ Although residents recognize IP communication skills as essential for their daily practice, they indicated that formal IP training was lacking. This implies that our current post-graduate training in IP communication is suboptimal, despite the World Health Organisation (WHO),^9^ Accreditation Council for Graduate Medical Education (ACGME)^17^ and Interprofessional Education Collaborative (IPEC)^5^ calls for improved communication between healthcare providers across professions.

### Influence of the clinical environment on IP communication

The health care environment is a complex system that has changed dramatically over the past decades and has posed many challenges to effective IP communication. The increasing use of computers and the electronic order systems have reduced the need for face-to-face interactions. In addition, the health care system is influenced by health insurance reimbursement and growing pressures on limited resources, such as time. Physicians face the difficulty of justifying time for communication and collaboration,18 compromising the time devoted to communication.

### Comparison of the literature

Personal factors that impact IP communication in this study included fear and a lack of self-confidence. There is a body of literature on the confidence of nurses and its effect on IP behaviors and competencies.^19^^,^^20^ The presence of these factors among residents is less well studied. This study reveals that even among senior residents, fear and self-confidence play a significant role in resident IP communication. Fear and low self-confidence can be exacerbated by systems culture, inadequate training, and the lack of feeling supported by the team. Support of the team contributes to the building of self-confidence, and team members feel better validated in their decisions when the team as a whole agrees.^18^ Working in developing relationships is one recommendation to enhance nurse-physician communication.^21^ Our study demonstrates that residents feel personal connections enhance working conditions;  however, residents struggle to maintain these connections in the setting of frequent rotating schedules. Finally, the influence of hierarchal boundaries and power-differentials on relationship building and IP communication persists, and is consistent with the existing literature.^22^^,^^23^

### Implications of the study for educational practice

Our study has several implications for IP education. Traditionally, residents are often trained in isolation from other healthcare professionals. Educational formats should include other professionals, consistent with the ‘Centre for the Advancement of Interprofessional Education’ (CAIPE) definition of IP training as occurring “when two or more professionals learn with, from and about each other to improve collaboration and the quality of care”.^24^ In order to change residents’ collaborative behavior in practice, a shift in the format and focus of training needs to take place, moving from individual to collaborative and team-based training. Interprofessional education can be embedded in training curricula using formats such as IP simulations, IP case discussions, and IP reading clubs, all suggested by the residents in this study.

### Implications of the study for systems changes

Based on residents’ perceptions described in this study, a few lessons can be learned to create changes in the system that can positively impact IP communication. An overview of the working IP team, including names and photos of the team members, can be displayed in the clinical environment, facilitating closed loop communication.^25^ The ability to practice closed-loop communication is essential in ad hoc teams performing acute resuscitative care under pressure, whether in the ED or any other clinical environment.

Another potential system change is to improve understanding of non-physician professionals’ roles and to establish trans-professional mentoring dialogues between nursing staff and new residents. Alignment of clinical schedules between nurses and residents can be considered to foster relationship building and end-of-shift debriefing. Finally, consideration should be paid to transitioning the current professional clinical rewards system from individual task achievement to high-level, collaborative, team-based IP performance.

### Limitations

This study provides insight into EM residents’ perceptions of IP communication in an academic clinical learning environment. Methods to increase the validity of the results included validation of the questioning route by independent EM educators, use of an independent focus group moderator, the use of iterative data collection until thematic saturation was reached, iterative data coding and analysis, and member checking of the resulting data themes.

Although our results resonate with the existing literature, their external validity and generalizability are limited since as the current study was performed in a single academic training center. The study focused on an ED setting. Therefore, some of the themes identified may not be generalizable to other post-graduate training settings and other hospitals in which IP teams and communication are the basis for best patient care practices. Other clinical learning environments may have other IP communication themes that were not prevalent in the emergency department setting. Residents working on inpatient wards may experience other types of barriers that are not captured in our results. In addition, nurses were not included in the focus groups since the study was designed to focus on resident-perceived barriers to resident IP communication to make recommendations for training interventions. Additional focus groups including non-physician professionals will add valuable perspectives to our understanding of IP communication in the ED. Last, the third focus group’s data collection time allotment was limited due to logistical constraints, lasting only twenty minutes, which is significantly shorter than the others groups.  Since the session revealed new complementary data, it was incorporated into the analysis.

## Conclusions

The objectives of the current study were to identify barriers and enablers of interprofessional (IP) communication, investigate current residency training in IP collaborative practice, and provide recommendations for improving training in IP communication to address current needs.  This study reveals that multiple barriers to effective IP communication in an academic ED exist in the clinical learning environment. These barriers are partly inherent in the system, but can be exacerbated by a lack of formal training in IP communication skills. The current post-graduate training in IP communication was found suboptimal, despite the WHO,^9^ ACGME^17^ and IPEC^5^ calls for improved IP communication. Residents indicated that IP communication is learned informally but could be explicitly enhanced by a variety of specific curricular interventions.  Further study in other clinical learning environments is needed to understand the common barriers to IP communication, as well as those unique to specific clinical environments, to inform curricular innovation.

### Acknowledgements

We would like to thank the residents from the Harvard Affiliated Emergency Medicine Residency for participating in the study. This study was supported by a grant from the Catharine van Tussenbroek Fund.

### Conflict of Interest

The authors declare that they have no conflict of interest.

## Supplementary material

Supplementary file 1 Appendix A. Questioning route to guide the discussion
