# Supplementary material for: Interprofessional communication in the emergency department: residents’ perceptions and implications for medical education
Source: Int J Med Educ. 2018 Oct 25;9:262–70. doi: 10.5116/ijme.5bb5.c111 (PMC6387781; doi:10.5116/ijme.5bb5.c111)
Supplement: Supplementary file 1 — Appendix A. Questioning route to guide the discussion [file ijme-9-262-S1.pdf]

Appendix A.

Questioning route to guide the discussion

1. Think back to the time when you first came into practice as a medical student or intern: Can you share some of your impressions or observations of communication between professionals?
2. What contributes to effective communication with other non-physician professions?
3. What are the main barriers to effective communication with other non-physician professionals?
4. How have you learned to communicate with members of a team?
5. Which learning experiences have had the greatest impact?
6. Do you feel you were adequately prepared to work in teams?
7. How can institutions best prepare residents to effectively communicate with other non-physician-professionals?
